# Supplementary material for: Persistence comparison of two Shiga-toxin producing Escherichia coli (STEC) serovars during long-term storage and thermal inactivation in various wheat flours
Source: PLoS One. 2024 Mar 8;19(3):e0299922. doi: 10.1371/journal.pone.0299922 (PMC10923466; doi:10.1371/journal.pone.0299922)
Supplement: S1 File — (DOCX) [file pone.0299922.s001.docx]

Table S1.

|  |  | All-Purpose Bleached | All-Purpose Unbleached | Bread | Self-Rising | Whole Wheat |
| --- | --- | --- | --- | --- | --- | --- |
| Amino Acids | **Analyte** | **Concentration (% [w/w])** | | | | |
|  | Alanine | 0.27 | 0.28 | 0.32 | 0.26 | 0.41 |
|  | Arginine | 0.40 | 0.40 | 0.47 | 0.40 | 0.64 |
|  | Aspartic Acid | 0.35 | 0.36 | 0.42 | 0.32 | 0.56 |
|  | Cysteine | 0.29 | 0.28 | 0.34 | 0.28 | 0.36 |
|  | Glutamic Acid | 3.04 | 3.10 | 3.68 | 2.82 | 3.84 |
|  | Glycine | 0.37 | 0.37 | 0.43 | 0.36 | 0.55 |
|  | Histidine | 0.28 | 0.27 | 0.24 | 0.24 | 0.29 |
|  | Hydroxyproline | 0.01 | 0.01 | 0.01 | 0.01 | 0.01 |
|  | Isoleucine | 0.31 | 0.31 | 0.36 | 0.29 | 0.41 |
|  | Leucine | 0.64 | 0.65 | 0.74 | 0.60 | 0.83 |
|  | Lysine | 0.26 | 0.33 | 0.35 | 0.31 | 0.38 |
|  | Methionine | 0.15 | 0.16 | 0.17 | 0.15 | 0.19 |
|  | Phenylalanine | 0.43 | 0.44 | 0.52 | 0.41 | 0.57 |
|  | Proline | 1.29 | 1.35 | 1.64 | 1.22 | 1.68 |
|  | Serine | 0.43 | 0.44 | 0.51 | 0.41 | 0.57 |
|  | Threonine | 0.24 | 0.23 | 0.27 | 0.22 | 0.33 |
|  | Tryptophan | 0.10 | 0.11 | 0.12 | 0.09 | 0.16 |
|  | Tyrosine | 0.26 | 0.27 | 0.32 | 0.25 | 0.34 |
|  | Valine | 0.34 | 0.34 | 0.39 | 0.32 | 0.48 |
|  | Total | 9.45 | 9.69 | 11.29 | 8.95 | 12.59 |
| Fatty Acids | **Analyte** | **Concentration (g/100g)** | | | | |
|  | Cis-Monounsaturated | 13.64 | 15.14 | 13.82 | 13.79 | 17.89 |
|  | Cis-Polyunsaturated | 62.68 | 61.43 | 62.25 | 62.93 | 60.20 |
|  | Saturated | 23.62 | 23.35 | 23.84 | 23.22 | 21.85 |
|  | Trans | 0.06 | 0.08 | 0.08 | 0.06 | 0.06 |
| Minerals | **Analyte** | **Concentration (ppm [w/w])** | | | | |
|  | Aluminum | 2.30 | 1.80 | 2.00 | 815.00 | 3.30 |
|  | Antimony | 0.01 | 0.01 | 0.01 | 0.01 | 0.01 |
|  | Arsenic | 0.01 | 0.01 | 0.01 | 0.01 | 0.01 |
|  | Barium | 2.10 | 2.11 | 1.78 | 2.36 | 4.60 |
|  | Beryllium | 0.01 | 0.01 | 0.01 | 0.01 | 0.01 |
|  | Bismuth | 0.02 | 0.02 | 0.02 | 0.02 | 0.02 |
|  | Boron | 0.30 | 0.50 | 0.20 | 1.00 | 0.70 |
|  | Cadmium | 0.04 | 0.04 | 0.04 | 0.04 | 0.04 |
|  | Calcium | 195.00 | 182.00 | 172.00 | 1970.00 | 340.00 |
|  | Chromium | 0.03 | 0.04 | 0.04 | 0.09 | 0.09 |
|  | Cobalt | 0.01 | 0.01 | 0.01 | 0.01 | 0.01 |
|  | Copper | 1.39 | 1.43 | 1.70 | 1.26 | 4.14 |
|  | Iron | 48.50 | 52.40 | 49.30 | 47.00 | 37.70 |
|  | Lead | 0.01 | 0.01 | 0.01 | 0.01 | 0.02 |
|  | Lithium | 0.10 | 0.10 | 0.10 | 0.10 | 0.10 |
|  | Magnesium | 268.00 | 289.00 | 304.00 | 325.00 | 1350.00 |
|  | Manganese | 8.97 | 8.91 | 7.92 | 9.87 | 35.10 |
|  | Mercury | 0.01 | 0.01 | 0.01 | 0.01 | 0.01 |
|  | Molybdenum | 0.26 | 0.27 | 0.24 | 0.28 | 0.61 |
|  | Nickel | 0.09 | 0.10 | 0.10 | 0.12 | 0.23 |
|  | Phosphorus | 982.00 | 1030.00 | 1110.00 | 4680.00 | 3010.00 |
|  | Potassium | 1460.00 | 1440.00 | 1410.00 | 1550.00 | 3480.00 |
|  | Selenium | 0.20 | 0.40 | 0.30 | 0.20 | 0.80 |
|  | Silver | 0.02 | 0.02 | 0.02 | 0.02 | 0.02 |
|  | Sodium | 7.00 | 6.00 | 8.00 | 12400.00 | 10.00 |
|  | Strontium | 0.84 | 0.95 | 0.81 | 6.78 | 2.33 |
|  | Thallium | 0.01 | 0.01 | 0.01 | 0.01 | 0.01 |
|  | Thorium | 0.10 | 0.10 | 0.10 | 0.10 | 0.10 |
|  | Tin | 0.01 | 0.01 | 0.01 | 0.01 | 0.01 |
|  | Titanium | 0.80 | 0.78 | 0.68 | 2.93 | 1.81 |
|  | Uranium | 0.01 | 0.01 | 0.01 | 0.04 | 0.01 |
|  | Vanadium | 0.01 | 0.01 | 0.01 | 0.02 | 0.01 |
|  | Zinc | 6.43 | 6.42 | 8.94 | 7.38 | 25.50 |
|  | Zirconium | 0.10 | 0.10 | 0.10 | 0.10 | 0.10 |
| Organic Acids | **Analyte** | **Concentration (% [w/w])** | | | | |
|  | Acetic Acid | 0.03 | 0.06 | 0.03 | 0.02 | 0.11 |
|  | Butyric Acid | 0.01 | 0.01 | 0.01 | 0.01 | 0.01 |
|  | Citric Acid | 0.02 | 0.02 | 0.01 | 0.01 | 0.08 |
|  | Fumaric Acid | 0.01 | 0.01 | 0.01 | 0.01 | 0.01 |
|  | Lactic Acid | 0.01 | 0.01 | 0.01 | 0.01 | 0.01 |
|  | Malic Acid | 0.01 | 0.01 | 0.01 | 0.01 | 0.01 |
|  | Tartaric Acid | 0.01 | 0.01 | 0.01 | 0.01 | 0.01 |
| Sugars | **Analyte** | **Concentration (% [w/w])** | | | | |
|  | Fructose | 0.25 | 0.25 | 0.25 | 0.25 | 0.25 |
|  | Glucose | 0.25 | 0.25 | 0.25 | 0.25 | 0.43 |
|  | Lactose | 0.25 | 0.25 | 0.25 | 0.25 | 0.25 |
|  | Maltose | 5.11 | 9.08 | 5.98 | 5.78 | 4.16 |
|  | Sucrose | 0.25 | 0.32 | 0.30 | 0.31 | 0.79 |


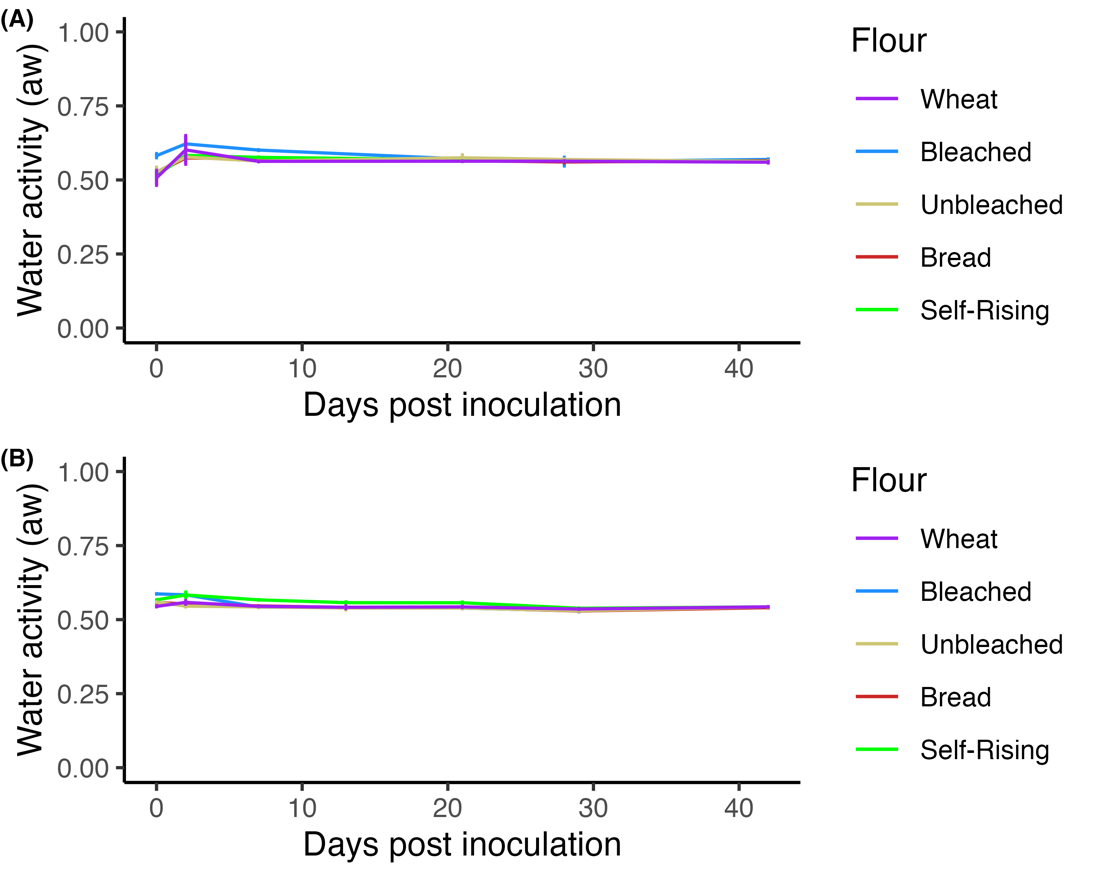


**Figure S1. Water activity for inoculated flours during storage.** The following wheat flour types were used: whole wheat (purple), all-purpose bleached (blue), all-purpose unbleached (yellow), bread (red), and self-rising (green). Two independent flour samples containing 1 g each were analyzed for water activity on each sampling day.
